# Vivisection: An Appeal to Reason

**Published:** 1892-10-29

**Authors:** 


### The Hospital

An Institutional Journal of

### Science, Medicine, Nursing, and Philanthropy.

*For Hospitals, Asylums, Infirmaries, Dispensaries, Medical Practitioners, Students, Nurses, and the Charitable Public.*

Vol. XIII.—No. 318.] [Oct. 29, 1892.

### Vivisection: An Appeal to Reason.

The medical man of the present day is a man of culture, of reason, and of conscience. Scientific studies make for reasonableness, and they make for truthfulness and simplicity of mind. The members of the medical profession have no contempt for the opinions of other classes of thinking and sober-minded persons. On the contrary, study has taught them how inadequate an instrument the single human mind may be, and how just and wise a result may be arrived at by the patient co-operation of many minds. Modern medicine, the medicine of the laboratory and of the scientific clinique, makes its appeal to the "common sense of most," just as the new astronomy did in its dawn; and, shall we not say, just as the new theology is doing at the present time?

We have long been anxious to convince right-minded persons of the non-medical world that most of the Englishmen who make experiments upon living animals are men of high character, of worthy motives, and of truly humane feeling. We have also striven to show that such experiments have not only been of great service in the arts of medicine and surgery, but have constituted a fundamental and important part of the scientific progress of the times. The recent heated controversy at the Church Congress, and the peculiar challenges put forward in the daily newspapers by Mr. Lawson Tait, Dr. Edward Berdoe, and others, have given rise to much aggravated feeling, and they have also left in some minds the impression that leading medical men, though strong in their resolution to stand by experimental physiology, are either unable or unwilling to give the public the means of forming a sound judgment upon this burning question.

It is well known, however, that medical men are both able and willing to help the public to understand the real merits of the controversy. It is true that Sir Andrew Clark, Sir James Paget, and others have declined to discuss the subject, by means of letters to the daily newspapers, with Mr. Lawson Tait and Mr. Berdoe. Surely every thoughtful and self-respecting man must see that this is the only possible decision! Mr. Lawson Tait himself cannot but be aware how futile and inconclusive such a discussion must have been. Indeed it is inexplicable how a man of Mr. Tait's knowledge and experience could have thought of proposing such a discussion. Is it possible to believe that science can be advanced by public gladiatorial combats between opposed scientists? The very temper of science itself is altogether antagonistic to such a method of procedure. When men contend with one another, and especially if they contend in public, each strives for the victory over his fellow—and human nature cannot do otherwise. But among true scientists the victory of individuals is not so much as thought of—each strives for the victory of the truth, for its clearer presentation, for its convincing and perfect demonstration. How then can true and self-respecting men of science range themselves on opposite sides, and each fight for his own hand in a public newspaper? It is impossible.

In our judgment it is profoundly important that the public, who have the power of the vote in our democratic country, should understand the true bearings of this question. We therefore ventured to ask Sir Andrew Clark, the distinguished and trusted President of the Royal College of Physicians, to definitely indicate, for the guidance of reasoning persons, what actual progress in physiological science and in the medical and surgical arts might properly be attributed to experiments upon living animals? That Sir Andrew feels deeply the injustice with which physiologists have been attacked, and the absolute necessity of the properly-regulated continuance of physiological experiments, is obvious from his reply, which we print in another place.

It will be seen, on reference to Sir Andrew Clark's notes, that he places experimental physiology on the highest conceivable ground. It is the will of the Creator, he contends, that the lower animals shall serve the legitimate purposes of the higher; "vicarious sacrifice is a law of the physical, mental, moral, and spiritual life." This is the only logical position which an intelligent and moral being can take up. To take any lower is to give the case away. The physiological experimenter who takes Sir Andrew Clark's position feels that he is serving God and man in the best way he knows when he is searching for new and useful truth in the quiet and silence of his laboratory.

Are physiological experiments, competently performed, "cruel"? Sir Andrew Clark says they are not; and he draws for readers the true distinction between cruelty, and pain legitimately inflicted for worthy ends. Many readers will be grateful for this clearness of thought and reasoning. All of us feel that vivisection is a painful necessity. The animal suffers; he suffers, not for himself, but for others; and he suffers, apparently, without hope of compensation. The human being who suffers vicariously has his reward in the inspiring hope of immortality. There is no immortality for the lower animals: so we think, and say. But who can tell us whether there be or not? May not the fact of the lower creature's vicarious sufferings for others and not for himself be the very sign and seal of his reward in some future state of existence—the title-deeds of his immortality? If wrong there be here, and in other uses to which men put animals, the High Divine Justice which will finally see all things righted, will not leave even the wronged animal outside the range of its universal retribution and redemption.

The question asked by the largest number of people concerning experiments upon living animals is this: Do they serve the absolute well-being of man? On this point Sir Andrew Clark's article is full and unanswerably convincing. According to Sir Andrew, the discovery of the circulation of the blood was to physiology what the discovery of the law of gravitation was to general science; and Harvey was the Newton of scientific medicine. Sir Andrew points out three departments of practical medicine and surgery which he considers owe either their origin or their present utility and value to experiments upon living animals. Those departments include the knowledge and management of infectious fevers, certain new and critical life-saving surgical operations, and the precise and scientific use of many of our most important drugs. For details the reader is referred to Sir Andrew's article itself; but we may here emphasise certain facts in modern medicine and surgery which of themselves almost constitute a new science of saving and prolonging life.

Ovariotomy may fitly be placed first among the new operations which have made the nineteenth century illustrious among all the centuries for the scientific progress of its medicine. Other operations, such, for example, as the removal of portions of the brain and spinal cord, the excision of parts of the stomach, the amputation of the larynx, the complete extirpation of one kidney, and the like, are not only to be numbered among the fruits of experimental physiology, but were never so much as dreamed of until experiments upon living animals had demonstrated their possibility and safety.

The vivisection controversy may be summed up in a paragraph: Is it cruel to experiment on animals? Is it wrong? Is it useless? If none of these questions can be answered in the affirmative; if, on the contrary, it can be proved that physiological experiments are not cruel, are not wrong, but are of the greatest possible service to men and animals, and are, in fact, the very life and soul of progressive medicine, then we must all try to be satisfied, and to give to physiologists the freedom and respect to which culture and character are everywhere entitled. On these three fundamental points of cruelty, morality, and utility Sir Andrew Clark is clear, convinced, and convincing. He makes it plain as noonday that throughout all nature the lower serves the higher, and that in the physiological laboratory the highest of all the services which animals can render to man and to one another is claimed from them. This conclusion is at least honest and rational: in our judgment it is also sound and final. It may, we think, at any rate be accepted provisionally until time and patient thought shall have thrown clearer and fuller light upon this difficult question.

